# Dimensional Stability of Polymer and Titanium Implant Scan Bodies After Repeated Steam Sterilization: A High-Resolution 3D Metrological In Vitro Study

**DOI:** 10.3390/jfb17050255

**Published:** 2026-05-20

**Authors:** Igor Smojver, Roko Bjelica, Yuval Reiser, Marko Vuletić, Vladimir Prpić, Dragana Gabrić

**Affiliations:** 1 Department of Oral Surgery, School of Dental Medicine, University of Zagreb, 10000 Zagreb, Croatia; ismojver@sfzg.hr (I.S.); rbjelica@sfzg.hr (R.B.); mvuletic@sfzg.hr (M.V.); 2 School of Dental Medicine, University of Zagreb, 10000 Zagreb, Croatia; yreiser@sfzg.hr; 3 Department of Dental Medicine, Clinical Hospital Centre Zagreb, 10000 Zagreb, Croatia; 4 Department of Fixed Prosthodontics, School of Dental Medicine, University of Zagreb, 10000 Zagreb, Croatia; vprpic@sfzg.hr

**Keywords:** computer-aided design, dental abutments, dental implants, in vitro techniques, polymers, sterilization, titanium

## Abstract

The increasing adoption of digital workflows in implant dentistry relies heavily on the accuracy of implant scan bodies (ISBs), which may be affected by repeated sterilization. This in vitro study evaluated the effect of 50 steam sterilization cycles on the dimensional stability of polymer and titanium ISBs. A total of 100 test specimens (*n* = 50 per material) were scanned before (T0) and after sterilization (T50) using a high-resolution intraoral scanner, generating 900 STL datasets for metrological analysis. Surface deviation, linear displacement, and angular deviation were assessed using validated industrial and dental software, with statistical evaluation performed through paired tests and linear mixed-effects models. Both materials exhibited statistically significant dimensional changes after sterilization (*p* < 0.001). Titanium scan bodies demonstrated greater linear deformation (69.76 μm) compared to polymer ones (49.50 μm), while maintaining superior angular stability (0.21° vs. −1.69° mean angular change in the polymer group). A significant interaction between material type and sterilization was observed. Despite high baseline precision, repeated autoclaving induced clinically relevant deviations in both materials. These findings indicate that cumulative sterilization cycles adversely affect ISB accuracy and highlight the importance of adhering to manufacturer recommendations to ensure optimal prosthetic outcomes.

## 1. Introduction

The integration of digital technology into implant dentistry has transformed prosthetically driven treatment planning. It allows precise data acquisition, manipulation, and fabrication of implant-supported restorations using intraoral scanners and computer-aided design/computer-aided manufacturing (CAD/CAM) systems [1]. Digital workflows simplify clinical procedures, reduce the number of patient visits, enhance communication between clinicians and dental laboratories and minimize errors commonly associated with traditional impression techniques [1,2]. The broad adoption of these technologies has made digital implant prosthodontics standard practice in modern clinical dentistry [1].

Implant scan bodies (ISBs) play an essential role in the scanning process because they function as reference components that accurately digitize the three-dimensional spatial position and orientation of the implant from the patient’s oral cavity [3]. The geometry of the ISBs is detected by intraoral scanners and then aligned with a corresponding digital library file in CAD software [4]. This matching process enables precise superimposition and the virtual reconstruction of the implant platform within the digital model [4]. The choice, design and proper scanning of ISBs directly impact the trueness and precision of digital impressions, which in turn affect the fit and performance of the final prosthetic restoration [5,6].

ISBs used in digital implant workflows are manufactured from two primary materials: polyetheretherketone (PEEK), a high-performance thermoplastic polymer, and titanium, a biocompatible metal [7]. Each material possesses distinct physical, mechanical, and optical properties that influence clinical performance. Titanium scan bodies show higher trueness and better scan ability than polymer scan bodies when captured using intraoral scanners, while polymer scan bodies exhibit higher precision in terms of reproducibility [7].

In clinical practice, scan bodies are frequently reused across multiple patients to reduce costs and environmental impact [8]. Reuse necessitates sterilization between patients, with steam autoclaving being the most common method employed in dental settings [8,9]. However, repeated exposure to high temperatures and pressure during autoclaving may induce dimensional changes in scan body materials, potentially compromising the accuracy of digital impressions [8,9,10]. Previous studies have demonstrated that autoclave sterilization has a measurable impact on scan body deformation, with polymer scan bodies showing greater susceptibility to dimensional changes compared to titanium [8,9].

Despite the clinical importance of ISB dimensional stability, substantial knowledge gaps remain [7]. Variations in study methodologies limit the generalizability of findings, and the comparative performance of PEEK polymer, used in this study as the Anatomic Healing Abutment (AHA) polymer scan body, and titanium ISBs under standardized high-cycle sterilization with current-generation scanners has yet to be fully clarified [7,8,9].

The aim of this controlled in vitro study was to evaluate the effect of 50 steam sterilization cycles on polymer and titanium scan bodies mounted on a standardized implant platform under clinically relevant conditions, with the null hypothesis that titanium scan bodies provide superior structural stability compared to the polymer scan bodies.

## 2. Materials and Methods

### 2.1. Study Design

This controlled in vitro study assessed the dimensional stability and scanning accuracy of PEEK polymer, using Straumann^®^ Anatomic Healing Abutment XC (AHA) (Institut Straumann AG, Basel, Switzerland), and titanium ISBs (Institut Straumann AG, Basel, Switzerland) before and after repeated sterilization, using a hierarchical data structure in which scans were nested within specimens.

### 2.2. Experimental Model

**Figure 1:**
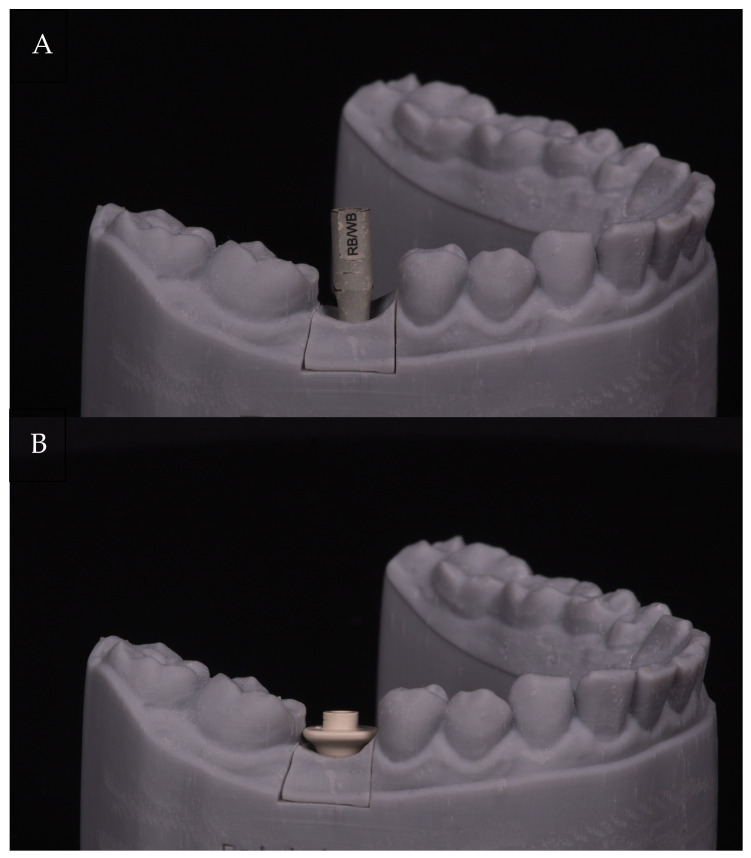
Standardized experimental setup showing the three-dimensional-printed mandibular model with implant scan bodies mounted on the implant analog (RB platform). (**A**) Titanium scan body. (**B**) AHA polymer (PEEK) scan body. Both configurations were used under identical conditions for all scanning procedures.

All measurements were performed on a three-dimensional-printed model derived from a real clinical case involving a single missing mandibular molar (Figure 1). The model was created from a high-resolution STL dataset and included a digital implant analog corresponding to a Straumann^®^ BLC implant, Regular Base (RB) platform (Institut Straumann AG, Basel, Switzerland), positioned in the molar region.

To ensure standardized implant geometry, a corresponding Straumann^®^ laboratory analog (RB platform) was embedded within the printed model. Both AHA polymer healing abutments/scan bodies and titanium scan bodies compatible with the Straumann^®^ BLC RB platform were used.

### 2.3. Specimens and Sample Size

A total of 102 physical specimens were included in the study. The test groups consisted of 50 AHA polymer healing abutments/scan bodies and 50 titanium scan bodies (*n* = 50 per material). Each specimen was evaluated both before sterilization (T0) and after 50 steam sterilization cycles (T50). In addition, two specimens (one AHA polymer and one titanium) were used as non-sterilized controls to assess scanner-related variability independent of material effects. To minimize potential confounding from manufacturing variations, all AHA polymer and titanium specimens were sourced from identical commercial production batches.

### 2.4. Sterilization Protocol

Sterilization was performed in a W&H Lisa steam sterilizer (W&H Sterilization S.r.l., Brusaporto, Italy) using the packaged instrument (wrapped load) cycle at 132 °C, with a 4-min holding phase and full drying cycle. Each specimen underwent 50 consecutive sterilization cycles as per group allocation.

The use of 50 sterilization cycles was intended to simulate an accelerated aging condition and represent a worst-case clinical scenario exceeding manufacturer recommendation, thereby allowing evaluation of cumulative thermomechanical effects on scan body stability.

### 2.5. Scanning Protocol

All scans were acquired using a fifth-generation 3Shape intraoral scanner (3Shape A/S, Copenhagen, Denmark). Scanning was performed by a single calibrated operator under standardized environmental conditions (temperature 23 ± 1 °C, relative humidity 50 ± 5%) to eliminate inter-operator variability [11]. Prior to data collection, the operator completed calibration sessions with repeated reference scans, as recommended by Revilla-León et al. [12].

Each test specimen was scanned three times at T0 and three times at T50 (6 scans per specimen). Given 100 test specimens, this resulted in 600 STL datasets. For control specimens, 50 independent scanning sessions were conducted per material, with three scans obtained per session (150 scans per control specimen). With two control specimens, 300 additional STL datasets were generated. Overall, 900 STL datasets were included in the final metrological analysis. Although only one physical control specimen per material was utilized, the extensive repeated measurements provided robust statistical power to accurately quantify and isolate scanner and operator variability independently of material deformation.

### 2.6. Dimensional and Metrological Analysis

Three-dimensional superimposition and data analysis were conducted following established digital evaluation protocols [8] to ensure high-resolution detection of deformations. Alignment of the STL datasets was achieved using a best-fit algorithm restricted exclusively to the stable, non-sterilized geometries of the printed model and the base of the implant analog. By strictly excluding the scan body surfaces from the alignment calculation, software overfitting was prevented, ensuring that the recorded deviations represented true material deformation.

**Figure 2:**
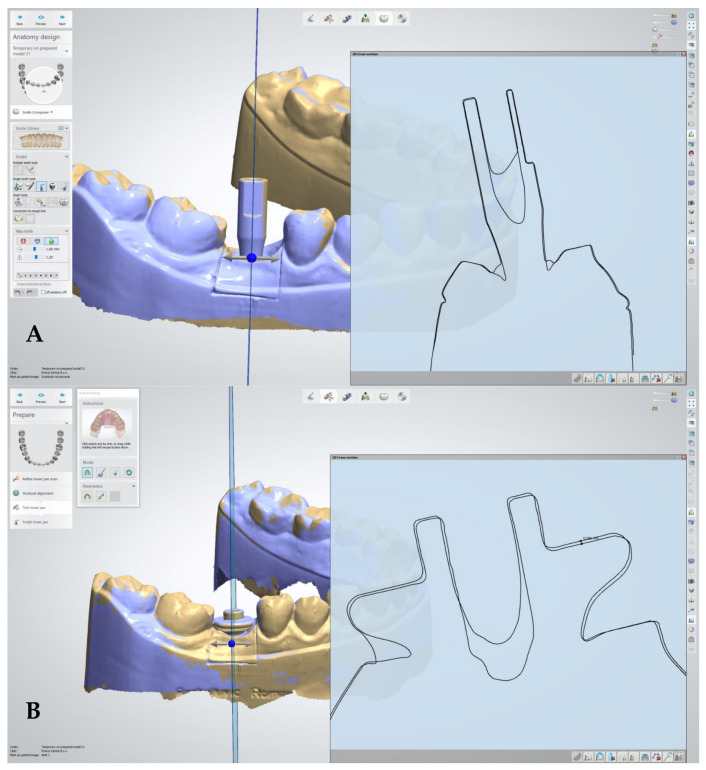
Linear deformation analysis of implant scan bodies following repeated sterilization. (**A**) Titanium scan body: two-dimensional linear measurement protocol along the implant axis performed using 3Shape Dental Designer, illustrating the reference geometry and evaluation of axial displacement. (**B**) Polymer (AHA) healing abutment/scan body: corresponding linear measurement protocol demonstrating axial displacement assessment under identical analytical conditions.

**Figure 3:**
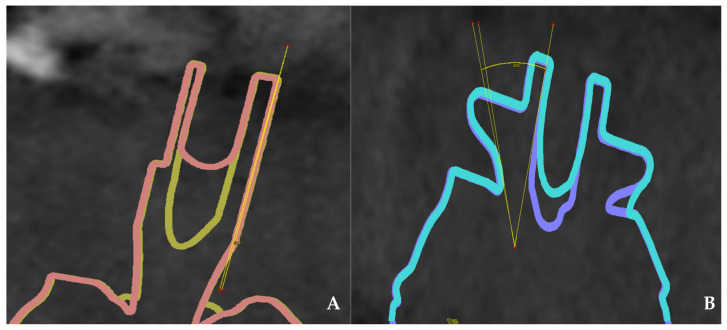
Angular deviation analysis of implant scan bodies following repeated sterilization. (**A**) Titanium scan body: evaluation of angular deviation using coDiagnostix^®^ software version 10.9.6, showing the reference axis and calculation of inclination changes relative to the baseline dataset. (**B**) Polymer (AHA) healing abutment/scan body: corresponding angular deviation analysis illustrating changes in scan body orientation after sterilization.

The metrological protocol included the following analytical steps:

- Surface Deviation: The primary outcome measure was the root mean square (RMS) surface deviation (µm), calculated using 3Shape Dental Designer (3Shape A/S, Copenhagen, Denmark).
- Linear Deformation (Figure 2): For the 2D analysis of linear deformation and the measurement of linear displacement along the implant axis, 3Shape Dental Designer was utilized.
- Angular Deviation (Figure 3): Secondary outcomes concerning angular stability and changes in the inclination of the scan bodies relative to the reference dataset were analyzed using coDiagnostix^®^ (Dental Wings Inc., Montreal, QC, Canada).
- Comprehensive Evaluation: In addition to these metrics, mean absolute deviation was assessed to provide a complete overview of the dimensional stability of AHA polymer and titanium specimens after 50 steam sterilization cycles.

### 2.7. Statistical Analysis

#### 2.7.1. Power Analysis

The power calculation was based on paired comparisons between T0 and T50, with a significance level of α = 0.05 and a statistical power of 1 − β = 0.80. Assuming a moderate standardized effect size (Cohen’s d = 0.50), a minimum of 34 specimens per group was required. By including 50 specimens per material group, the study achieved over 95% power to detect moderate effects and approximately 80% power to detect small-to-moderate effects (d ≈ 0.40).

#### 2.7.2. Statistical Modeling

Data distribution for all linear (3Shape) and angular (coDiagnostix^®^) measurements was assessed using Shapiro–Wilk tests and graphical inspection. For the linear mixed-effects models, the assumptions of normality, independence, and homoscedasticity were validated by evaluating residual plots prior to final analyses.

- Primary Comparisons: Within-material changes (T0 vs. T50) for RMS, linear displacement, and angular deviation were analyzed using paired *t*-tests or Wilcoxon signed-rank tests where normality assumptions were violated.
- Hierarchical Modeling: To account for the three repeated scans per specimen and the nested data structure, linear mixed-effects models were fitted. Fixed effects included material (AHA polymer vs. titanium), sterilization status (T0 vs. T50), and their interaction, with random intercepts for each specimen.
- Reproducibility: Measurement reliability across scanning sessions and software analyses was quantified using intraclass correlation coefficients, coefficients of variation, and Bland–Altman analysis.
- Significance: Effect sizes (Cohen’s d and partial η^2^) were reported with 95% confidence intervals. Multiplicity was controlled using the Holm–Bonferroni correction, and statistical significance was defined as *p* < 0.05.

## 3. Results

### 3.1. Linear Dimensional Stability

The high-resolution metrological analysis demonstrated distinct patterns of dimensional changes across the experimental groups. All 100 test specimens (50 AHA polymer, 50 titanium) successfully underwent 50 steam sterilization cycles without any catastrophic material failure. Summary values for linear deformation, calculated as the mean of three independent scans per specimen, are presented in Table 1.

### 3.2. Quantitative Comparative Analysis

At the baseline (T0), both materials exhibited negligible surface variability, with mean linear dimensions of 1.86 ± 0.13 μm for AHA polymer and 1.66 ± 0.12 μm for titanium, confirming high initial manufacturing precision. These baseline values represent deviation from the reference CAD model and reflect the intrinsic trueness of the scanning system rather than physical deformation of the specimens. Following 50 sterilization cycles (T50), both groups demonstrated statistically significant increases in linear deformation (*p* < 0.001).

### 3.3. Statistical Modeling and Interaction Effects

The linear mixed-effects model (LMM) revealed a highly significant interaction between material type and sterilization status (*p* < 0.001). Both materials were affected by thermomechanical stress, but titanium scan bodies exhibited greater mean linear deformation (69.76 μm) compared to AHA polymer scan bodies (49.50 μm) at T50.

Analysis of the LMM results highlighted both the main effects of sterilization and the interaction between material type and sterilization, as detailed below:

- Time Effect: The cumulative impact of 50 sterilization cycles was the primary contributor to dimensional change (Coefficient: 47.64 μm; z = 35.35, *p* < 0.001).
- Interaction Effect: Titanium scan bodies showed an additional 20.47 μm of linear displacement relative to the AHA polymer group following sterilization (*p* < 0.001).
- Effect Size: Cohen’s d indicated an extremely large magnitude of change for both materials (AHA d = 3.12; Titanium d = 4.50), suggesting that although AHA polymer is more stable, neither material is entirely resistant to cumulative thermomechanical alterations.

**Figure 4:**
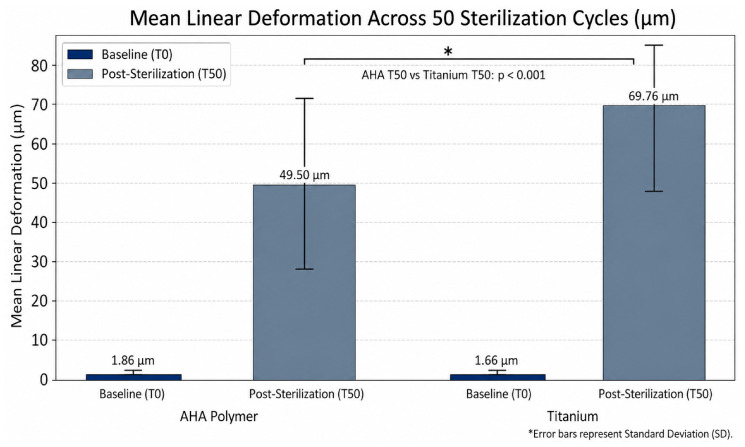
Comparative analysis of mean linear deformation (μm) between AHA polymer and titanium scan bodies at baseline (T0) and after 50 steam sterilization cycles (T50). Error bars represent the standard deviation (SD). An asterisk (*) denotes a statistically significant difference between material groups at T50 (*p* < 0.001), as determined by the linear mixed-effects model.

Contrary to the initial hypothesis that titanium scan bodies would provide superior structural stability, these results indicate that AHA polymer healing abutments/scan bodies maintained better linear dimensional stability over 50 sterilization cycles. The observed titanium deformation (69.76 μm) may exceed the threshold for passive fit in multi-unit implant restorations, whereas the AHA polymer’s performance suggests higher resilience to the cumulative 132 °C thermal cycles (Figure 4).

### 3.4. Angular Stability and Deviation

The angular stability of the scan bodies was evaluated using coDiagnostix^®^ software by measuring deviations in inclination (degrees, °) before and after 50 sterilization cycles. This angle reflects the orientation of the scan body reference surface relative to the implant axis, rather than a direct measure of material deformation, thereby explaining the higher absolute baseline values observed in the polymer group. Detailed results are summarized in Table 2.

### 3.5. Comparative Angular Analysis

At baseline (T0), the titanium group demonstrated remarkably high initial stability with a mean angular deviation of 0.02 ± 0.01°, while the AHA polymer group exhibited a higher baseline mean of 30.11 ± 0.39°. Following 50 sterilization cycles (T50), both materials showed statistically significant changes in their angular orientation (*p* < 0.001).

### 3.6. Statistical Modeling: Time and Material Interaction

The linear mixed-effects model (LMM) confirmed a highly significant interaction between the material and the sterilization process (*p* < 0.001).

- AHA Polymer Group: Sterilization led to a significant decrease in the mean angle (Coefficient: −1.69°; z = −71.59, *p* < 0.001). This “flattening” effect suggests a systematic thermomechanical deformation of the polymer structure over 50 cycles.
- Titanium Group: Conversely, titanium scan bodies showed a significantly smaller but consistent increase in angular deviation (Interaction Coefficient: +1.89° relative to the AHA trend; *p* < 0.001).
- Effect Magnitude: Cohen’s d calculations revealed a very large effect size for both materials (AHA d = −3.11; Titanium d = 1.89), confirming that repeated sterilization cycles fundamentally alter the geometric orientation of the scan body interface.

**Figure 5:**
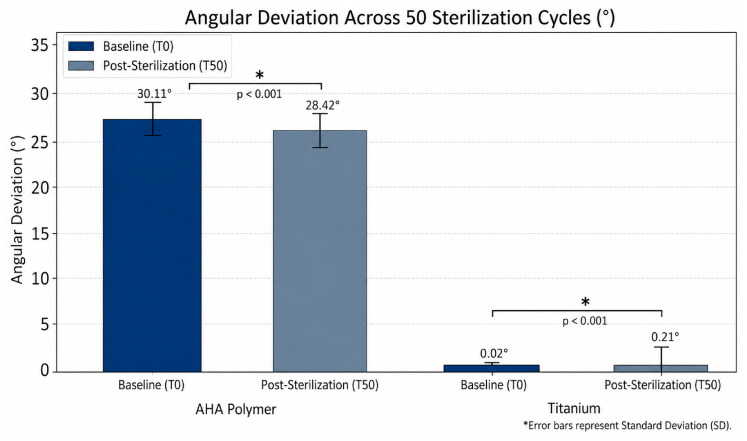
Comparison of mean angular deviation (°) for AHA and Titanium scan bodies. Note the different baseline scales, with Titanium showing superior absolute angular stability, while AHA polymer exhibits a significant systematic decrease in angle after 50 sterilization cycles (T50). Error bars represent Standard Deviation (SD).

The results highlight a distinct behavior between the two materials under thermal stress. While the titanium group maintained a significantly lower absolute angular deviation (0.21° at T50) compared to the AHA polymer group which experienced a more pronounced angular shift (−1.69° change). While titanium remained largely within this clinical threshold, the AHA polymer’s systematic shift suggests that its reuse as a scan body beyond a certain number of cycles may compromise the precision of the final prosthetic restoration (Figure 5).

## 4. Discussion

The findings of this study challenge the conventional assumption that titanium scan bodies provide superior dimensional stability compared to polymer alternatives. Following 50 steam sterilization cycles, titanium scan bodies exhibited greater mean linear deformation (69.76 μm) than AHA polymer scan bodies (49.50 μm), with both materials demonstrating statistically significant dimensional changes (*p* < 0.001). This behavior may be attributed to differences in material properties between the materials, including the lower elastic modulus of PEEK, which may facilitate more effective stress redistribution and partial elastic recovery, as well as differences in coefficient of thermal expansion and stress relaxation under repeated thermal cycling [13,14].

These results have important implications for clinical practice and the development of standardized reuse protocols.

### 4.1. Interpretation of Linear Dimensional Changes

Notably, the mean linear deformation observed in titanium scan bodies (69.76 μm) and AHA polymer (49.50 μm) approached values that may have clinical implications for prosthetic fit. Although no definitive clinical threshold for acceptable misfit has been established, Abdelrehim et al.’s [15] systematic review concluded that the current literature provides inadequate data to determine a universal threshold, noting that vertical misfit up to 1 mm and horizontal misfit up to 345 μm were associated with no adverse outcomes in some studies.

However, these thresholds are largely derived from conventional workflows, whereas modern digital implant workflows require substantially higher precision. In this context, deviations in the range observed in the present study (approximately 50–70 μm) may still be clinically relevant, particularly in multi-unit restorations where passive fit is critical.

Our findings contrast with previous studies evaluating different sterilization protocols. Costa Santos et al. [8] reported that both titanium and PEEK polymer scan bodies remained below 50 μm deviation after 100 autoclaving cycles, although PEEK polymer showed greater deformation, particularly at the abutment level. Similarly, Kato et al. [9] found significant differences in distance and angle when comparing scan bodies before and after autoclave treatment but importantly noted that repeated connection and disconnection alone did not substantially impact deformation. The discrepancy between our results and prior literature may be attributed to differences in cumulative thermomechanical stress exposure and measurement methodology.

Diker et al. [16] demonstrated that sterilization generally increased PEEK polymer scan body displacements, with titanium showing lower displacements than PEEK polymer under most conditions. However, their protocol involved only 25 sterilization cycles, suggesting that the relationship between material stability and cycle number may not be linear.

Taken together, these findings underscore that variations in sterilization protocols, cycle numbers, and measurement techniques may substantially influence material behavior, thereby limiting direct comparability across studies.

### 4.2. Angular Stability Consideration

The angular stability analysis revealed distinct material-specific behaviors under thermal stress. Titanium maintained superior absolute angular stability, but the AHA polymer demonstrated a systematic “flattening” effect that may compromise prosthetic precision. Rutkunas et al. [17] demonstrated that horizontal misfits were less tolerated than vertical ones and may be more detrimental to implant–prosthesis fit, with angular deviations contributing to framework misfit in multi-unit restorations. Another study further demonstrated through finite element analysis that an angular misfit as small as 0.083° can produce bone loading up to 20 MPa in the cortical layer, indicating that even minimal angular deviations, which cannot be detected clinically, may lead to substantial peri-implant bone stress [18].

Collectively, these findings suggest that the systematic angular shift observed in AHA polymer scan bodies may exceed clinically acceptable thresholds, potentially compromising passive fit and inducing biomechanical stress in multi-unit implant-supported restorations.

### 4.3. Manufacturer Guidelines and Clinical Implications

These findings must be interpreted within the context of manufacturer recommendations. Straumann^®^ explicitly recommends single use only for the AHA polymer, while for scan bodies, a maximum of 10 sterilization cycles is advised. Our study, which extended to 50 cycles, demonstrates that exceeding these recommendations results in clinically significant dimensional alterations.

Previous findings support manufacturer guidelines, although results on reuse and sterilization remain mixed, likely due to differences in study methodologies. One study reported that reuse of scan bodies up to 10 times did not consistently affect the accuracy of partially dentate multi-implant dental casts, whereas others demonstrated that repeated use and sterilization beyond manufacturer guidelines can lead to significant deformation and reduced accuracy in both conventional impressions and digital scans, prompting recommendations to limit reuse [10,19,20].

### 4.4. Biological and Clinical Implications

Beyond dimensional stability, the microbiological implications of scan body reuse warrant critical consideration. Barreiros et al. [21] demonstrated that approximately 30% of retrieved healing abutments showed remnant biofilm biomass even after standard cleaning and autoclave sterilization protocols.

This biofilm persistence presents a dual clinical concern. Firstly, even when sterilization achieves microbial kill, the organic biofilm matrix and residual endotoxins may remain trapped within material micropores. It was confirmed that while autoclave sterilization reduced bacterial load, surface analysis revealed areas of debris and material accumulation on abutments following various decontamination protocols [22]. Secondly, Ichioka et al. [23] demonstrated that no treatment modality resulted in complete biofilm removal from implant surfaces, even when combining mechanical and chemical decontamination methods.

Each subsequent cleaning and sterilization cycle contributes to progressive surface alterations. The relationship between surface roughness and biofilm formation is well established in the literature, with studies showing that increased surface roughness and surface free energy promote biofilm formation on dental implant and abutment surfaces [24,25,26,27].

Even when sterilization achieves microbial elimination, the residual organic matrix and bacterial components may still exert biological activity. It has been demonstrated that, compared to periodontitis, peri-implantitis is characterized by a more extensive inflammatory infiltrate, a stronger innate immune response, greater tissue destruction, and a faster rate of progression [27]. Furthermore, residual materials may contribute to adverse host responses, as highlighted by the synergistic effect between foreign body reactions and biofilm remnants [28].

Clinically, the combined effect of dimensional alterations and incomplete biofilm decontamination suggests that strict adherence to single-use or highly limited reuse protocols is paramount to prevent errors in final prosthesis fabrication. Future research should prioritize in vivo clinical trials to assess the true intraoral performance of reused scan bodies, alongside advanced microbiological analyses to quantify pathogenic biofilm retention deep within the micro-topography of sterilized polymer and titanium components.

### 4.5. Study Limitations

This study was conducted under controlled in vitro conditions that may not fully replicate the clinical environment, including the effects of saliva, blood, and mechanical stresses during intraoral scanning. First, the use of a single missing mandibular molar model limits the generalizability of the findings to more complex clinical scenarios, such as full-arch rehabilitations or angulated implant placement. While this design maximized internal validity by isolating material-specific deformations, future studies should evaluate these scan bodies in diverse anatomical setups. The sterilization protocol utilized a single autoclave type and cycle parameters. Variations in sterilization equipment (different autoclave classes or brands) and specific cycle profiles (heating and cooling rates) alter the thermomechanical stress applied to the materials and may yield different dimensional outcomes. Furthermore, the single-implant-analog design does not account for the cumulative scanning errors and deviations that occur across multiple implants in partially or fully edentulous arches. While utilizing a single calibrated operator maximized internal validity and eliminated inter-operator error for precise metrological assessment, it inevitably limits the external validity of the study, as it does not reflect the operational variability present in routine clinical practice. Additionally, the study evaluated dimensional changes but did not assess the biological consequences of residual biofilm or surface degradation on peri-implant tissue response. Future studies should incorporate clinical validation and long-term follow-up to determine the prosthetic outcomes associated with scan body reuse.

## 5. Conclusions

Within the limitations of this in vitro study, polymer (AHA) scan bodies demonstrated higher linear dimensional stability (49.50 μm deformation compared to 69.76 μm for titanium), whereas titanium scan bodies exhibited greater resistance to angular deviation (0.21° change versus a −1.69° systematic shift in the polymer group) after 50 steam sterilization cycles. Therefore, the null hypothesis that titanium provides superior structural stability was partially rejected. The clinically relevant dimensional changes observed in both materials emphasize that repeated cumulative sterilization compromises ISB accuracy. Strict adherence to manufacturer reuse guidelines is highly recommended to ensure the precision and passive fit of digital implant-supported restorations.

## Figures and Tables

**Table 1:** Descriptive statistics for linear deformation (μm) before (T0) and after (T50) 50 steam sterilization cycles.

Study Group	Time Point	N (Specimens)	Mean ± SD (μm)	Range (Min–Max)	95% CI
**AHA Polymer**	T0	50	1.86 ± 0.13	1.60–2.20	[1.82, 1.90]
	T50	50	49.50 ± 21.57	4.00–86.00	[43.37, 55.63]
**Titanium**	T0	50	1.66 ± 0.12	1.43–1.97	[1.62, 1.69]
	T50	50	69.76 ± 21.42	39.00–112.00	[63.68, 75.84]

**Table 2:** Angular deviation (°) before (T0) and after (T50) 50 steam sterilization cycles.

Study Group	Time Point	N (Specimens)	Mean ± SD (°)	Range (Min–Max)	95% CI
**AHA Polymer**	T0	50	30.11 ± 0.39	28.99–30.70	[30.00, 30.22]
	T50	50	28.42 ± 0.66	26.99–29.61	[28.23, 28.60]
**Titanium**	T0	50	0.02 ± 0.01	0.01–0.03	[0.018, 0.022]
	T50	50	0.21 ± 0.14	0.00–0.50	[0.17, 0.25]

## Data Availability

The original contributions presented in this study are included in the article. Further inquiries can be directed to the corresponding author.

## Abbreviations

The following abbreviations are used in this manuscript:

CAD/CAM	Computer-Aided Design/Computer-Aided Manufacturing
ISBs	Implant Scan Bodies
PEEK	Polyetheretherketone
AHA	Anatomic Healing Abutment
RB	Regular Base
RMS	Root Mean Square
LMM	Linear Mixed-Effects Model
